# Supplementary material for: Empirical methods for controlling false positives and estimating confidence in ChIP-Seq peaks
Source: BMC Bioinformatics. 2008 Dec 5;9:523. doi: 10.1186/1471-2105-9-523 (PMC2628906; doi:10.1186/1471-2105-9-523)
Supplement: Additional File 1 — A variety of html documents from the USeq web site detailing the available applications, their best usage, output file type descriptions, command line menus, etc. [file 1471-2105-9-523-S1.zip › USeqUserGuides/JavaDoc.zip!/JavaDoc/gata/aligner/Alignment.html]

Alignment


|  |  |  |
| --- | --- | --- |
| **Overview** \| **Package** \| **Class** \| **Use** \| **Tree** \| **Deprecated** \| **Index** \| **Help** | |  |
| **PREV CLASS**   **NEXT CLASS** | **FRAMES**    **NO FRAMES**     **All Classes** |  |
| SUMMARY: NESTED \| FIELD \| CONSTR \| METHOD | DETAIL: FIELD \| CONSTR \| METHOD |  |


---


## gata.aligner Class Alignment

```
java.lang.Object
  gata.aligner.Alignment
```

**All Implemented Interfaces:**: java.io.Serializable, java.lang.Comparable

---

``` public class Alignment extends java.lang.Object implements java.io.Serializable, java.lang.Comparable ```

**Author:**
:   nix
    Class to represent a windowed sub alignment, a portion of a BLAST local alignment.
    A serialize array of these are saved and made availible for the GATAPlotter Program.

**See Also:**: Serialized Form

---

**Constructor Summary**

`Alignment(java.lang.String s1,
int start1,
int stop1,
java.lang.String s2,
int start2,
int stop2,
int aScore,
int ori,
AlignParams ap,
LocalAlignment la)`


| **Method Summary** | |
| --- | --- |
| `int` | `compareTo(java.lang.Object otherObject)` |
| `void` | `drawAlignment(java.awt.Graphics2D g2)`             Called every time the alignPanel is repaint() ed |
| `java.lang.String` | `getAlignmentString()` |
| `double` | `getBitScore()` |
| `int[]` | `getCoordinates()` |
| `int` | `getOrientation()` |
| `java.lang.String` | `getParentLocAlignString()` |
| `int[]` | `getRelativeCoordinates()` |
| `int` | `getScore()` |
| `java.lang.String` | `getSeq1()` |
| `java.lang.String` | `getSeq2()` |
| `java.util.ArrayList` | `getShapes()` |
| `boolean` | `isCompVisible()` |
| `void` | `makeShapes(double refX, double refY, double compX, double compY, double ntLenBig, double widthC, double heightD)` |
| `void` | `printAlignment()` |
| `void` | `setCompVisible(boolean compVisible)` |
| `void` | `setSeqs(java.lang.String s1, java.lang.String s2)` |

**Methods inherited from class java.lang.Object**

`equals, getClass, hashCode, notify, notifyAll, toString, wait, wait, wait`

**Constructor Detail**

### Alignment

```
public Alignment(java.lang.String s1,
                 int start1,
                 int stop1,
                 java.lang.String s2,
                 int start2,
                 int stop2,
                 int aScore,
                 int ori,
                 AlignParams ap,
                 LocalAlignment la)
```


**Method Detail**

### getScore

```
public int getScore()
```

---


### getBitScore

```
public double getBitScore()
```

---


### getOrientation

```
public int getOrientation()
```

---


### getCoordinates

```
public int[] getCoordinates()
```

---


### getShapes

```
public java.util.ArrayList getShapes()
```

---


### getSeq1

```
public java.lang.String getSeq1()
```

---


### getSeq2

```
public java.lang.String getSeq2()
```

---


### setSeqs

```
public void setSeqs(java.lang.String s1,
                    java.lang.String s2)
```

---


### drawAlignment

```
public void drawAlignment(java.awt.Graphics2D g2)
```

:   Called every time the alignPanel is repaint() ed

---


### printAlignment

```
public void printAlignment()
```

---


### getAlignmentString

```
public java.lang.String getAlignmentString()
```

---


### getParentLocAlignString

```
public java.lang.String getParentLocAlignString()
```

---


### makeShapes

```
public void makeShapes(double refX,
                       double refY,
                       double compX,
                       double compY,
                       double ntLenBig,
                       double widthC,
                       double heightD)
```

---


### compareTo

```
public int compareTo(java.lang.Object otherObject)
```

:   **Specified by:**: `compareTo` in interface `java.lang.Comparable`

---


### getRelativeCoordinates

```
public int[] getRelativeCoordinates()
```

---


### setCompVisible

```
public void setCompVisible(boolean compVisible)
```

---


### isCompVisible

```
public boolean isCompVisible()
```


---


|  |  |  |
| --- | --- | --- |
| **Overview** \| **Package** \| **Class** \| **Use** \| **Tree** \| **Deprecated** \| **Index** \| **Help** | |  |
| **PREV CLASS**   **NEXT CLASS** | **FRAMES**    **NO FRAMES**     **All Classes** |  |
| SUMMARY: NESTED \| FIELD \| CONSTR \| METHOD | DETAIL: FIELD \| CONSTR \| METHOD |  |


---
